# Does conservative or arthroscopic management of canine medial coronoid disease lead to improved outcomes?

**DOI:** 10.18849/ve.v10i1.700

**Published:** 2025-02-04

**Authors:** Anant Andy Banerjee

**Keywords:** arthroscopy, canine, conservative management, dogs, elbow dysplasia, medial coronoid disease

## Abstract

**PICO question:**

In dogs diagnosed with medial coronoid disease, does arthroscopic surgical intervention, compared with conservative management, result in improved mobility and reduced pain?

**Clinical bottom line:**

**Category of research:**

Treatment.

**Number and type of study designs reviewed:**

Three studies were identified directly addressing the PICO question. One paper was a retrospective cohort study, another study was a prospective randomised control trial, and the third was a non-randomised non-blinded observational study.

**Strength of evidence:**

Weak.

**Outcomes reported:**

The first study was a prospective non-randomised study that did not identify a difference in gait evaluation at the 52 week recheck in dogs treated conservatively compared to those treated arthroscopically, with lameness exacerbated in the arthroscopic treatment group until the 26 week recheck. The second study, a retrospective non-randomised cohort study, showed greater, but not statistically different, owner-reported clinical metrology scores in dogs treated arthroscopically compared to those treated conservatively. Liverpool Osteoarthritis in Dogs (LOAD) and Pain Severity Scores (PSS) were higher but not statistically significant, yet Pain Interference Scores (PIS) were statistically significantly higher at 52 weeks in arthroscopically treated dogs compared to conservatively treated dogs. Age at diagnosis and at time of questionnaire completion were statistically significant for LOAD, PSS, and PIS, with older dogs having higher scores. The third study performed a non-blinded observational study assessing canine patients with bilateral medial coronoid disease, with unilateral arthroscopic subtotal coronoidectomy performed on the most clinically affected limb. Radiographs and computed tomography (CT) imaging were performed at diagnosis, with radiographs taken at follow-up. At the time of follow-up, arthroscopically treated limbs had a higher radiographic score than those treated conservatively, although a significant improvement in lameness was seen at the walk in arthroscopically treated limbs. Conservatively managed dogs showed an unchanged (non-significant) gait. Radiographic changes did not appear to correlate to severity of clinical signs.

**Conclusion:**

The quality of the published evidence available to answer the PICO question is weak, due to the design of the three reviewed studies. Low patient populations in these three studies also hinder the statistical power of any recommendations made. None of the three studies assesses the complex nature of medial coronoid disease to clearly answer the question posed. The decision to recommend arthroscopy over conservative management therefore depends on the judgement and experience of the veterinary surgeon attending the case. Additionally, assessment of the imaging findings is important when discussing prospective treatment options.

## How to apply this evidence in practice

The application of evidence into practice should take into account multiple factors, not limited to: individual clinical expertise, patient’s circumstances and owners’ values, country, location or clinic where you work, the individual case in front of you, the availability of therapies and resources.

Knowledge Summaries are a resource to help reinforce or inform decision-making. They do not override the responsibility or judgement of the practitioner to do what is best for the animal in their care.

### Clinical scenario

A 9-month-old male entire Labrador presents to your practice with a 2-month history of intermittent left thoracic limb lameness. Physical examination reveals a 2/5 left thoracic limb lameness with pain localised to the elbow joint. Computed tomography (CT) of the elbow joints is recommended which shows an in situ apical to radial incisure fissure of the medial coronoid process with no apparent radioulnar incongruity of the left elbow joint. You discuss two treatment options with the owners: either conservative management with analgesia, a moderated activity regime and physical therapies, or arthroscopic assessment of the joint and fissure removal. The owners ask which treatment option is most likely to lead to reduced pain as well as a return to normal function. You want to be able to provide the most efficacious treatment option to the owner.

### The evidence

Three studies were identified from literatures searches performed on PubMed and CAB Abstracts that answered the PICO. The first study was a prospective randomised control trial (Burton et al., 2011). The second was a retrospective cohort study (Dempsey et al., 2019). The third was a non-blinded observational study (Seidler et al., 2023) where patients were identified from radiographs and computed tomography taken retrospectively then followed prospectively. All three papers assessed the short- and medium-term effects of conservative management compared to arthroscopic management of medial coronoid disease in canine elbow joints. Burton et al. (2011) used objective force platform analysis. Dempsey et al. (2019) used owner-reported outcome measures for assessment of differences between the treatment groups. Seidler et al. (2023) used a combination of radiographic assessment, CT assessment, owner-reported outcome measures, and subjective gait analysis. None of the studies could demonstrate major clinically significant differences between treatment outcomes between conservative or arthroscopic management.

### Summary of the evidence

**Burton et al. (2011):** Conservative versus arthroscopic management of medial coronoid process disease in dogs: a prospective gait evaluation **Aim: **Prospectively evaluate conservative management versus arthroscopic treatment of medial coronoid disease over a 52 week period.

Population	Client owned dogs of any breed, sex, and body weight presenting to Langford Veterinary Services, University of Bristol, and Dick White Referrals, Suffolk. Eligibility criteria for inclusion: Dogs less than 2 years of age at time of diagnosis. Unilateral forelimb lameness localised to the elbow joint on clinical examination. Complete clinical examination to deem healthy other than unilateral forelimb lameness. Bilateral orthogonal shoulder and elbow radiographs with signs consistent of unilateral medial coronoid disease. Radiographs assessed as per the International Elbow Working Group (IEWG) guidelines for different degrees of osteophytosis. Criteria for exclusion: Dogs that did not exactly meet inclusion criteria.
Sample Size	20 dogs.
Intervention Details	Conservative Management (CM); 9/20 dogs: Dogs discharged with a 6 week course of tepoxalin at 10 mg/kg orally once daily. Post-diagnosis exercise regime of room rest for one week, then 5 minute lead exercise twice daily the following week. Exercise increased by 5 minutes per week thereafter for two months. A gradual return to normal activity was then advised as per the owner's discretion. Arthroscopic Treatment (AT); 11/20 dogs: Arthroscopy was performed by medially placed portals with a 2.4 mm 30 degree fore oblique arthroscope. Fibrillated coronoid apices were identified with evidence of chondromalacia in elbows based on the modified Outerbridge scoring system with no evidence of concurrent medial humeral condylar ‘kissing’ lesions or osteochondrosis dissecans (OCD) lesions. Coronoid fragments were removed by 2.0 mm arthroscopic forceps with an arthroscopic burr used to remove any locally fibrillated or chondromalacic cartilage. Joint lavage was performed and routine closure performed A compressive dressing was applied for 24 hours. Discharged with a 6 week course of tepoxalin at 10 mg/kg orally once daily. An identical post-operative exercise to dogs in the CM group was prescribed.
Study design	Prospective non-randomised study.
Outcome studied	Gait analysis by means of force platform assessments: All dogs had pre-treatment gait analysis. Assessment performed for the affected & unaffected limb. Gait analysis performed pre-treatment, then at 4 weeks, 8 weeks, 26 weeks, and 52 weeks post-treatment.
Main findings (relevant to PICO question)	7/20 dogs had a left forelimb lameness, 13/20 had a right forelimb lameness. 3/9 and 4/11 dogs with left forelimb lameness were in the CM and AT groups respectively, 6/9 and 7/11 dogs with right forelimb lameness were in the CM and AT groups respectively. 16/20 dogs were Labrador retrievers, 1 each of German shepherd, Airedale terrier, Staffordshire bull terrier, and border collie. Average body weight in the AT group pre-treatment was 30.3 kg and 33 kg at post-treatment, with the average body weight in the CM group being 27.9 kg at pre-treatment and 31.8 kg at post-treatment. No statistical difference was noted in pre-treatment or post-treatment body weight between the two groups. Lameness identified for 1–14 months prior to diagnosis, manifesting between 4.5–18 months of age. No difference in pre-treatment age of onset of lameness was noted. No significant difference identified in IEWG radiographic score between CM and AT groups. Dogs in CM group trotting slower than those in the AT group at 1 month postoperatively, which was statistically significant with a p-value of < 0.05. Trotting velocity recovered subsequently thus that at 26 and 52 weeks post-treatment, trotting velocities in both groups were faster than that of pre-treatment. Dogs in the AT group had the greatest asymmetry in total support motion at 8 weeks post-treatment, then improved to symmetry at 26 and 52 weeks. Significant effect of week (P < 0.001) but not treatment type on elbow moment (EM). Unaffected limb EM and moment asymmetry was greater in the AT group than the CM group (P = 0.0035). CM group also had a significant week effect (P = 0.006) with steady reduction in unaffected limb EM asymmetry from pre-treatment through to 52 weeks. Greatest difference in power asymmetry between affected and unaffected limbs noted at 4 and 8 weeks post-treatment, with power symmetry best at 52 weeks. Significant effect of treatment type on unaffected elbow eccentric power (P < 0.001) but no significant effect of week in treatment. Significant effect of time (P < 0.05) but not treatment type or interaction between treatment and week in the affected elbow. No therapeutic benefit of AT compared to CM for the 52 week evaluation. Lameness in AT group appeared exacerbated to the 26 week point in comparison to the CM group between affected and unaffected limbs.
Limitations	Not possible to tell if patients treated in CM were suffering from pathology of an equivalent nature or severity as the cartilage was not visualised. Small group sizes limiting statistical power; type II error. Velocity assesses whole dog movement, not individual dog movement. Removal of fragmented medial coronoid process (FMCP) does not address underlying disease pathology or other changes.

Table note...

**Dempsey et al. (2019):** A comparison of owner-assessed long-term outcome of arthroscopic intervention versus conservative management of dogs with medial coronoid process disease **Aim: **Evaluate owner-assessed long-term outcome of dogs with medial coronoid disease treated with arthroscopy or conservative management using a combined owner-reported outcome questionnaire.

Population	Client owned dogs of any breed, sex, and body weight presenting to the University of Liverpool Small Animal Teaching Hospital. Eligibility criteria for inclusion: Lameness localised to one or both thoracic limbs on subjective gait analysis. Orthopaedic evaluation revealed pain on elbow palpation. Combination of above. Computed tomography (CT) imaging of elbows supporting a diagnosis of medial coronoid disease (MCD). Complete data entry for each patient. Completed combined owner questionnaire for Liverpool Osteoarthritis in Dogs (LOAD) and Canine Brief Pain Inventory (CBPI). Criteria for exclusion: Dogs that did not exactly meet inclusion criteria Dogs that had died since their treatment for MCD. Patients assessed over an 8-year period.
Sample Size	67 dogs. Labrador retrievers were the most commonly reported breed (40/67 dogs) with German shepherd dogs (6/67) and Rottweilers (4/67) the next two most commonly reported breeds.
Intervention Details	Conservative Management (CM); 23/67 dogs: Weight reduction in dogs with body condition score of 6/9 or greater. Use of a non-steroidal anti-inflammatory drug for 6 weeks +/- paracetamol/codeine for 1–2 weeks. Increasing lead-restricted exercise for 8 weeks. Arthroscopic Intervention (AI); 44/67 dogs: Performed by board-certified surgeon or resident in-training under direct supervision. Medial portal placement with use of 2.4 mm 30 degree oblique arthroscope. Fragment removal only in 30/44 dogs; chondroplasty of the MCP only in 10/44 dogs and inspection only in 4/44 dogs. Post-operative assessments in person or by teleconsultation at 6 weeks. Discharge instructions given following surgery identical to those given to owners of canine patients within CM group.
Study design	Retrospective non-randomised cohort study.
Outcome studied	Primary efficacy outcome measure (objective): Liverpool Osteoarthritis in Dogs (LOAD) score (x/52). 13 questions scored from 0–4 awarded per question. Total score per limb interpreted as mild (0–10), moderate (11–20), severe (21–30) or extreme (31–52). Canine Brief Pain Inventory (CBPI) score; total CBPI score (x/100). Pain Severity Score (PSS) (x/40). Pain Interference Score (PIS) (x/60). Quality of Life (QoL) score = (x/44). The QoL score is a separate score within the CBPI, specifically aiding in bringing the owner’s emotion into the assessment of patient pain response. Secondary efficacy outcome measure (subjective): Age at time of questionnaire completion. Whether patients still receiving medication or not. Median average distance covered per day (miles). Whether dogs were exercised on or off lead. Whether gait at exercise was walking or trotting. Any limitations to a dog’s willingness to exercise. Correlation between scoring indices (LOAD and total CBPI, LOAD and PIS, LOAD and PSS).
Main findings (relevant to PICO question)	Population Assessments: Mean bodyweight was 31.2kg in the AI group (range 16.5–65.5 kg) and 31.1 kg in the CM group (range 24.3–33.9 kg); no statistical difference in weight between groups. Mean age at diagnosis was 17 months in the AI group (range 5–64 months) and 31 months in the CM group (range 6–81 months); no statistical difference in age was noted. Mean age at completion of questionnaire was 87.5 months in the AI group (range 24–148 months) and 63 months (range 25–124 months); no statistical difference in age was noted. Majority of dogs in AI group diagnosed with bilateral disease (34/44); 6 dogs had right elbow MCD, 4 dogs had left elbow MCD. Majority of dogs in CM group diagnosed with bilateral disease (16/23); 6 dogs had right elbow MCD, 1 dog had left elbow MCD. Median total CT score for affected elbows was 7/12 in both the AI and CM groups; no statistical difference identified. Primary efficacy outcome measure (objective): Median LOAD score for combined patient population was 14/52 (range 6–20). Median total CBPI score for combined patient population was 11/100 (range 1–27). Median LOAD/PSS/PIS score for AI group was 14/52, 4/40 and 5.5/60 respectively. Median LOAD/PSS/PIS score for CM group was 9/52, 3/40 and 3/60 respectively. Although median LOAD/PSS/PIS score was higher in AI group than CM group, linear regression showed no statistical difference (P = 0.066 for LOAD, P = 0.10 for PSS), but was statistically different for PIS (P = 0.028). Specific type of AI performed (inspection, chondroplasty, fragment removal) was not significant for LOAD, PIS, or PSS score. Secondary efficacy outcome measure (subjective): Dogs in the AI group were significantly older (P = 0.004) than those in the CM group at time of questionnaire completion. Larger proportion of dogs in the CM group were off lead (82.6% vs 77.3%) and displayed a more active gait (69.6% vs 63.6%) than those in the AI group. Larger proportion of dogs in CM group covering a greater median distance per day (2.5 miles vs 1.5 miles). No significant difference between AI/CM groups for average amount of exercise (P = 0.058), whether dogs were exercise on or off lead (P = 0.505) or whether exercise was mainly walking or more active (P = 0.547). Owners were the main limiting factor in their dog’s willingness to exercise in both groups (56.8% for AI, 65.2% for CM). Type of medication used for treatment at the time of questionnaire completion was not significantly associated with LOAD or PSS for both treatment groups but was statistically significantly associated with PIS scores (P = 0.016). Dogs on non-steroidal anti-inflammatory drugs (NSAIDs) (P = 0.033) or NSAIDs plus other drugs (P = 0.014) had lower PIS scores. QoL in the AI group was scored excellent (18/44), very good (18/44), and good (8/44). QoL in the CM group was scored as excellent (12/23), very good (6/23) and good (5/23). Linear regression did not identify any significant associations between LOAD, PSS or PIS and breed, sex or weight. Older age at diagnosis was significantly associated with higher LOAD, PSS, and PIS scores (P = 0.048, P = 0.026, and P = 0.046 respectively). Age at questionnaire completion remained significant in final multivariable analyses.
Limitations	No clinical metrology instrument (CMI) questionnaires completed at time of diagnosis. CMIs give an impression of a dog’s overall function, rather than specific information at the joint level. Retrospective nature of cases prevented randomisation of dogs, therefore selection bias possible; higher proportion of cases receiving arthroscopy vs conservative management. Small group size in CM group leads to type II error. Variability in surgical experience performing arthroscopy (board-certified surgeon vs resident in-training). No characterisation of a standardised arthroscopic grading system employed in AI group. Dogs in AI group significantly older when questionnaire performed; may have indicated bias for surgeons to choose to treat older dogs to treat conservatively vs arthroscopically at diagnosis.

Table note...

**Seidler et al. (2023):** Dogs with bilateral medial coronoid disease can be clinically sound after unilateral arthroscopic fragment removal—preliminary study **Aim: **Evaluate the outcome of conservative management as compared to arthroscopic subtotal coronoidectomy in a canine subject with bilateral medial coronoid disease.

Population	Client owned dogs of any breed, sex, and body weight presenting to the Small Animal Teaching Hospital at Tierärztliche Hochschule Hannover. Eligibility criteria for inclusion: Bilateral diagnosis of medial coronoid disease (MCD). One forelimb more clearly identifiably clinically affected than other limbs based on lameness and physical examination changes related to pathology; pain, crepitus, external rotation of forelimb, joint swelling. Had arthroscopy performed on the most clinically affected limb with other limb treated conservatively. Complete documentation of radiographs, computed tomography (CT) imaging, arthroscopic surgery report, and clinical examination findings. Complete data entry for each patient. Completed subjective gait assessment scores and owner questionnaires for Liverpool Osteoarthritis in Dogs (LOAD). Criteria for exclusion: Dogs that did not exactly meet the inclusion criteria. Dogs that had died since their treatment for MCD. Dogs with concurrent elbow joint pathology (ununited anconeal process, osteochondritis of the medial humeral condyle, flexor tendon enthesiopathy).
Sample Size	24 dogs included in study: Crossbreed dogs were the most commonly reported breed (9/24 dogs) with Labrador Retrievers (6/24) reported as the next most commonly reported breed. Each dog served as its own control, therefore 24 dogs with 48 elbows were assessed.
Intervention Details	Arthroscopic Surgery (AS); 24 elbows Performed by the same veterinary surgeon. Arthroscopic procedure was performed on the more clinically affected limb. Fragment removal was performed. Discharge instructions given following surgery identical to those given to owners of canine patients within CM group. Conservative Management (CM); 24 elbows Lead walking for 6 weeks. Use of a non-steroidal anti-inflammatory drug for 14 days. Supportive exercises including physiotherapy, aqua training, osteopathy or electrotherapy. In some cases, feed additives to support joint function (green-lipped mussel extract, devil’s claw, glucosamine, chondroitin) were additionally recommended.
Study design	Non-blinded observational study. Diagnosis was made based on retrospective assessment of data from radiographs and CT imaging. Prospective data collected from follow-up examinations.
Outcome studied	Primary radiographic outcome measurement (objective): Mediolateral flexed and caudocranial views of each elbow taken at diagnosis and following surgery and after conservative intervention during follow-up examination (average 32.5 months). Osteoarthritis evaluation performed according to International Elbow Working Group (IEWG) guidelines with modification to focus on size of osteophytes as an indicator of arthrosis. Scoring index from IEWG guidelines 0–3 based on severity. Trochlear notch sclerosis (TNS) quantification based on previously outlined methodology with TNS ratio (level of TNS: ulnar depth). Primary CT outcome measurement (objective): Parameters described included: Type of pathology present; single fragment, multiple fragment, fissures, combination of lesions, none of the above. Type of fragmented medial coronoid process (FMCP); fragment/fissure along radial incisure of ulna, fragmentation affecting apex of MCP, radial incisure-tip fragment/fissure or combination. Fragment dislocation (yes or no). Primary efficacy outcome measure (objective): LOAD score (x/52). 13 questions scored from 0–4 awarded per question. Total score per limb interpreted as mild (0–10), moderate (11–20), severe (21–30) or extreme (31–52). Secondary efficacy outcome measure (subjective): Subjective gait analysis with measurements taken twice at initial examination then twice at follow-up consultation. Correlational analysis: Correlational analysis between measurably parameters; modified IEWG, TNS, LOAD score, and degree of lameness.
Main findings (relevant to PICO question)	Mean bodyweight was 35.2 kg (range 10.5–68.5 kg). Mean age at diagnosis was 37.4 months (range 5–94 months). Mean age at follow-up was 70.9 months (range 20–151 months. Primary radiographic outcome measure (objective) before treatment: 12/24 elbows had modified IEWG score 0 in AS and CM groups. 3/24 elbows had modified IEWG score 0 in AS and 1 in CM. 3/24 elbows had modified IEWG score 0 in CM and 1 in AS. 1/24 elbows had modified IEWG score 0 in CM and 2 in AS. 1/24 elbows had modified IEWG score 1 in CM and 3 in AS. 1/24 elbows had modified IEWG score 2 in CM and 3 in AS. Primary radiographic outcome measure after treatment: 5/24 elbows in the CM group had the same IEWG score. 16/24 in AS group had a higher IEWG score after treatment VS before, where only 1/24 in the CM group had a higher score than before treatment. 20% of elbows in AS group had the same score after treatment compared to initial exam, no elbows improved after surgery, 80% had deterioration in IEWG score. 58% of elbows in CM group were unchanged in IEWG score, 42% had deterioration in IEWG score. Median TNS value increased by 0.04 mm in AS group after surgery with p-value of 0.022. Median TNS value increased by 0.05 mm in the CM group after treatment with p*-*value of 0.228. Primary CT outcome measure before treatment: FMCP size was quantified in 47/48 elbows. Single FMCP fragment identified in 18/24 of CM cases and 19/24 of AS cases; p-value of 0.629. 6/24 CM cases had a dislocated fragment whilst 13/24 AS cases had a dislocated fragment; p-value of 0.077. Fragment size quantified by CT was 0.185 cm^2^ in the AS group and 0.124 cm^2^ in the CM group; p-value of 0.660. Primary client-based questionnaire assessments (objective): Filled out by owners and sent back during follow-up period. Median LOAD score in CM group was 9/52, median LOAD score in AS group was 10/52. Mean LOAD score in CM group was 9.5/52, median LOAD score in AS group was 13/52; p-value of 0.003. AS treated limb has 75% higher or equal LOAD score compared to CM treated limb. Secondary efficacy outcome measure (subjective): 184 values from 24 dogs were available for analysis. 8 missing values due to incorrect documentation. 3% of elbows treated by CM showed no deterioration in lameness after treatment at walking; 30% for elbows treated by AS after treatment at walking. 50% of elbows treated by AS improved lameness score after treatment at walking, 20% deteriorated. 5% of elbows treated by CM showed no deterioration in lameness after treatment at trotting; 12.5% deteriorated. 35% of elbows treated by AS showed no deterioration in lameness after treatment at trotting; 30% improved, 35% deteriorated. Primary correlation analysis: All patients with a modified IEWG score 3 showed no lameness at walking at initial examination. Patients with modified IEWG score 2 had evenly distributed lameness score of 0 or 1 in gait at initial presentation. Patients with modified IEWG score 2 or 3 had evenly distributed lameness score of 0 or 1 at trot. Patients treated by AS had overall significantly higher lameness scores (p-value < 0.001) and modified IEWG radiographic scores (p-value of 0.02) compared to those treated by CM. Higher LOAD scores related to an increase in modified IEWG score. 8/20 elbows in the CM group with a dislocated fragment showed higher modified IEWG scores. 6/8 elbows had a larger than average fragment size. Elbows with a dislocated fragment showed a fragment larger than average with a deterioration in modified IEWG score (4/20).
Limitations	Non-controlled study thus results considered preliminary in nature. Accuracy and ability to compare arthroscopic treatment in one leg to conservative treatment in the contralateral limb. No measurement of obesity as body weight, not body condition score noted for patients. Small number of dogs (24) with low statistical power. Variable non-uniform follow-up examination period. Positive selection bias by performing arthroscopic procedure on clinically most affected limb. Positive bias given non-blinding of lameness scores. Query regarding validity of objective gait analysis from subjective gait assessment data gathered. No analysis of the effect of feed additives (though mentioned within treatment regime).

Table note...

### Appraisal, application and reflection

The PICO question is intended to be narrow in its focus. Whilst being the most commonly diagnosed pathology of elbow dysplasia, medial coronoid disease (MCD) is only one feature of this complex syndrome. There are a myriad of different pathologies within elbow dysplasia, including fragmentation of the medial coronoid process (FMCP), osteochondrosis dissecans (OCD) of the medial humeral condyle, elbow incongruity, and ununited anconeal process (UAP) (Vezzoni & Benjamino, 2021). There are a variety of different reported outcomes for surgery or conservative management for the varied pathological presentations of elbow dysplasia identified. Therefore, this narrow PICO question only addresses part of the complex treatment recommendations for dogs with elbow dysplasia. In addition, MCD itself can present in different ways and the treatment for these may differ. These different pathologies are poorly defined in most studies.

Analysis of the three papers (Burton et al., 2011; Dempsey et al., 2019; Seider et al., 2023) relating to the PICO question shows overall weak evidence to suggest arthroscopy is superior to conservative management for dogs with MCD.

Burton et al. (2011) demonstrated no therapeutic benefit of arthroscopic management of FMCP compared to conservative management in gait evaluation by 52 weeks. Lameness was exacerbated in the arthroscopically managed group up to 26 weeks compared to the conservatively managed group, then lameness was comparable between both groups at 52 weeks. The very small numbers of patients within both treatment groups (11 dogs treated arthroscopically versus 9 dogs treated conservatively) imbues an inherently low statistical power to this study. Whilst the clear strength of this study is its prospective nature, its non-randomised design and low patient populations are significant limitations, meaning that the possibility of findings being significant or non-significant is low.

Dempsey et al. (2019) performed a retrospective assessment of cases over 8 years looking at owner-reported outcomes using two clinical metrology instruments (CMI’s). No differences between the treatment groups could be identified on linear regression of the difference CMI’s performed. No power study was performed to assess the numbers of patients required to assess statistical differences. Additionally, the arthroscopic group contained a heterogenous population of different arthroscopic treatments performed (inspection alone, chondroplasty, fragment removal). The morbidity of each of these may not have been accounted for on result analysis given the low numbers of some of these treatments. Additionally, no LOAD or CBPI scoring was performed at the outset of each patient’s treatment, making it difficult to assess the effect of treatment on initial pre-treatment levels of lameness or pain. These factors may contribute to the lack of statistically significant findings within this study. Strengths of this study include the larger patient populations than the Burton et al. (2011) paper, use of validated outcome measures such as CMI’s and use of computed tomography (CT) to score the extent of elbow disease. The lack of a power study to assess patient population sizes for statistical significance is the main limitation of this study. Some of the results are close to statistical significance and may ultimately be clinically significant were patient populations large enough.

Seidler et al. (2023) used 24 dogs with bilateral MCD, using the less affected limb as a control to assess the effect of surgery on the most clinically affected limb. Whilst 80% of patients (19/24 elbows) treated with arthroscopy showed a deterioration in IEWG radiographic scoring, only 42% of conservatively managed patients (10/24) showed worsening in IEWG scoring. Subjective gait assessment was improved in 50% of dogs where arthroscopy was performed at walking with 30% showing improvement at trotting. Gait patterns in conservatively managed patients remained largely unchanged. Whilst Liverpool Osteoarthritis in Dogs (LOAD) scoring was helpful in assessing changes between treatment groups, the author of this Knowledge Summary review struggles to understand how clients could have effectively differentiated mobility differences between limbs using the LOAD questionnaire as each dog enrolled in the study in effect acted as its own control. No LOAD questionnaire was performed prior to the study commencing, making comparability of scores (to differentiate any differences in outcome before and after both treatment types) impossible. Lack of blinding was also noted in the clinician performing lameness assessments. Many of the dogs within the study were over 36 months in age, therefore spectrum of disease and comparability of findings to other studies, such as the Burton et al. (2011) paper, is challenging as patients in that study were less than two years of age. Arthroscopic treatment tended to show improvements in clinical gait pattern when walking despite radiographic findings showing deterioration in scores.

There were several papers within the literature search that could not be included in this review as they did not completely answer the PICO question. Burton (2023) recently published a literature review of arthroscopic treatments for the assessment and management of MCD in dogs. 9 papers were found that relate to removal of fragments in dogs with MCD. These include studies by Bouck et al. (1995), Burton et al. (2011), Dempsey et al. (2019), and Meyer-Lindeberg et al. (2003). Additional papers reviewed included Huibregtse et al. (1994), Theyse et al. (2000), Barthélèmey et al. (2014), Galindo-Zamora et al. (2014), and Read et al. (1990). Meyer-Lindenberg et al. (2003) suggested more favourable outcomes in dogs having arthroscopy (60.1% good outcome) compared to arthrotomy (45.6% good outcome) for FMCP. No direct comparison was performed in this paper with conservative management. When assessing overall improvement in lameness (good and satisfactory groups), there is no statistical difference in dogs treated by arthrotomy (73.7%) or arthroscopy (92.5%) for dogs with FMCPs alone (Meyer-Lindenberg et al., 2003).

Barthélèmey et al. (2014) identified improvement in gait analysis which approached significance when compared to pre-operative values in a prospectively assessed group of 15 dogs having arthroscopic management of MCD by a mixture of fragment removal, subtotal coronoidectomy, and ulnar osteotomy. Galindo-Zamora et al. (2014) identified an improvement in kinetic variables following arthroscopy in a group of 14 dogs with unilateral MCD treated with arthroscopic removal and subchondral bed burring. Both studies lack a control population, therefore their comparability to patients treated conservatively need to be carefully considered.

Whilst papers directly comparing conservative to arthroscopic management are reported within the Burton (2023) review, some papers using prospective gait analysis documented some improvement in function versus pre-treatment lameness. Additionally, many of these papers do not contain a control population of patients treated with conservative or non-surgical management, which makes comparison of the available data very challenging. Also, not all studies have had equivalent diagnostic imaging performed, with some being assessed radiographically and others having had CT performed. It is for these reasons specifically that this review article was not included within the analysed papers relating to the PICO question. Other studies have documented the relative success of arthroscopic management for dogs with MCD (Cruz & Mason, 2022; Garnier et al., 2023; Lahiani et al., 2023) but are prone to type II error with low patient numbers and a lack of control population make the assessment of the results gathered makes application of this information difficult. Interestingly though, a ECVS survey of 132 specialist surgeons with over 1200 years of surgical experience showed that the majority of specialists recommend arthroscopic FMCP removal in a juvenile dog with focal elbow dysplasia with a high chance of functional improvement (Farrell et al., 2018), which seems to contradict the current evidence base.

Interpretation of meta-analysis results performed by Evans et al. (2008) must be undertaken with caution, given the inconsistencies in the papers assessed at that time regarding conservative management techniques and lameness scoring systems. A more recent meta-analysis of the available evidence was performed by Kähn et al. (2023). Data from relevant studies was selected via the Preferred Reports Items of Systematic Reviews and Meta-Analyses (PRISMA) guidelines and extracted with subsequent analysis by three meta-analyses: success rate, mean difference and standardised mean difference. 14/494 papers were identified including many of the previously mentioned papers, and the Burton et al. (2011) and Dempsey et al. (2019) papers critically appraised by the author. Standardised mean difference meta-analysis was used due to observer bias from the two other meta-analyses employed, confirming that no statistical differences were identifiable between medical and surgical therapies.

Therapeutic elbow arthroscopy itself is a procedure with known morbidity and does not fully resolve lameness in all dogs. Second-look arthroscopic in dogs following previous arthroscopic subtotal coronoidectomy (Coppieters et al., 2016) on average 2.2 years following arthroscopic treatment showed a number of additional pathologies such as medial compartment cartilage erosion. Perry & Li (2014) identified a major complication rate of 4.8% (36/750) and minor complication rate of 10.7% (76/750) of cases in a retrospective assessment of complications in 750 arthroscopically managed cases.

CMIs have been increasingly used in veterinary medicine to provide validated means of assessing clinical outcomes. CMIs achieve this by providing scoring metrics which can then be statistically assessed by means of client-based observational questionnaires. Since the initial documented validity of LOAD, by Hercock et al. (2009), multiple validated CMIs have been introduced to veterinary medicine for both dogs and cats (Benito et al., 2013; Muller et al., 2016) in a variety of languages (Alves et al., 2022; Ragetly et al., 2019). LOAD and Canine Brief Pain Inventory (CBPI) CMIs are used the most frequently. The LOAD questionnaire is a validated CMI generating a score out of 52 based on 13 questions, assessing mobility in general and also when exercising. The CPBI is a two-part CMI generating a pain severity score (PSS) out of 40 from four questions graded from 0–10 and a pain interference score (PIS) out of 60 from six questions graded similarly (Brown et al., 2007). An additional overall quality of life score (QoL) is rated on a five-point scale categorised from poor to excellent. Regardless of the scoring system used, higher CMI scores have been shown to be associated with a greater degree of osteoarthritis and potentially worse outcomes (Alves et al., 2022). The use of LOAD and CBPI provides useful data within the Dempsey et al. (2019) study, yet the low patient population in both study groups underpowers the statistical relevance of the data analysed. Recall bias is noted with CMI use in medium- to long-term follow-up analysis (Pappa et al., 2023), and therefore prospective analysis of CMIs should be considered. The design of the Dempsey et al. 2019 study retrospectively analysing LOAD and CPBI at 12 months following treatment with no scoring performed at date of treatment commencement could therefore succumb to recall bias. Further studies assessing the impact of conservative versus arthroscopic management of canine MCD should prospectively use a CMI from the date of commencing treatment and stratifying outcomes subsequently over time.

Assessment of outcomes after conservative versus arthroscopic management of MCD needs to account for the spectrum of disease identified within affected elbow joints. Studies to date do not complete account for this. Consequently, it is not yet possible to provide a conclusive answer to the PICO question as to whether arthroscopic surgical intervention results in improved mobility and reduced pain when compared with conservative management for dogs with MCD. Careful consideration of signalment, physical examination findings, and the results of diagnostic imaging should be made prior to discussing treatment recommendations with owners on a case-by-case basis. Given the lack of high-quality statistically significant data, strong recommendations for arthroscopic management of MCD cannot currently be made. Based on the evidence described above with the methodological weaknesses and low patient populations of the three studies relevant to this PICO question (Burton et al., 2011; Dempsey et al., 2019; Seider et al., 2023), clinicians could therefore consider treating these cases with a period of conservative management. Arthroscopy would therefore then be reserved for cases refractory to conservative management. The merits and disadvantages of arthroscopy based on the available literature should be discussed with owners at the time of diagnosis so an informed decision can be made.

### Methodology

**Table 2:** Caption placeholder

	Search strategy
Databases searched and dates covered	CAB Abstracts on the OVID interface; date of coverage 1920–May 2024 PubMed accessed via the NCBI website; date of coverage 1920–May 2024
Search terms	Cab Abstracts: (dog or dogs or bitch* or canine*) (elbow dysplasia or ED or developmental elbow disease or medial coronoid disease or MCD) (arthroscop* OR arthrotom*) 1 and 2 and 3 Pubmed: (dog or dogs or bitch* or canine*) (elbow dysplasia or ED or developmental elbow disease or medial coronoid disease or MCD) (arthroscop* OR arthrotom*) 1 and 2 and 3
Dates searches performed:	24 May 2024

Table note placeholder

**Exclusion / Inclusion Criteria:** 

Exclusion	Studies not identified within the specified inclusion quidelines. Duplicates. Not relevant to species or condition. Papers in other language other than English. Did not answer the PICO question directly or partially answered.
Inclusion	Retrospective and prospective studies comparing arthroscopy with conservative management directly or comparing arthroscopy with arthrotomy. Review articles summarising available literature.

Table note placeholder

**Search Outcome:** 

Database	Number of results	Excluded –duplicates	Excluded – not relevant to species or condition	Excluded – in language other than English	Excluded – did not answer the PICO question directly	Excluded– partly answers the PICO question	Total relevant papers
CAB Abstracts	88	1	0	22	61	4	0
PubMed	89	2	2	1	73	8	3
Total relevant papers when duplicates removed							3

Table note placeholder

### Acknowledgements

The author would like to thank Clare Boulton (Head of Library and Knowledge Services, RCVS Knowledge) for assisting with the literature sources and refining search terms.

### ORCiD

Anant Andy Banerjee: https://orcid.org/0009-0008-2145-2408

### Conflict of Interest

The author declares no conflicts of interest.

## Disclaimer

Knowledge Summaries are a peer-reviewed article type which aims to answer a clinical question based on the best available current evidence. It does not override the responsibility of the practitioner. Informed decisions should be made by considering such factors as individual clinical expertise and judgement along with patient’s circumstances and owners’ values. Knowledge Summaries are a resource to help inform and any opinions expressed within the Knowledge Summaries are the author’s own and do not necessarily reflect the view of the RCVS Knowledge. Authors are responsible for the accuracy of the content. While the Editor and Publisher believe that all content herein are in accord with current recommendations and practice at the time of publication, they accept no legal responsibility for any errors or omissions, and make no warranty, express or implied, with respect to material contained within. For further information please refer to our Terms of Use.
